# Supplementary material for: Development of Flame-Retardant Polylactic Acid Formulations for Additive Manufacturing
Source: Polymers (Basel). 2024 Apr 10;16(8):1030. doi: 10.3390/polym16081030 (PMC11053787; doi:10.3390/polym16081030)
Supplement: Supplementary file 1 [file polymers-16-01030-s001.zip › polymers-2938686-supplementary.pdf]

Supplementary information

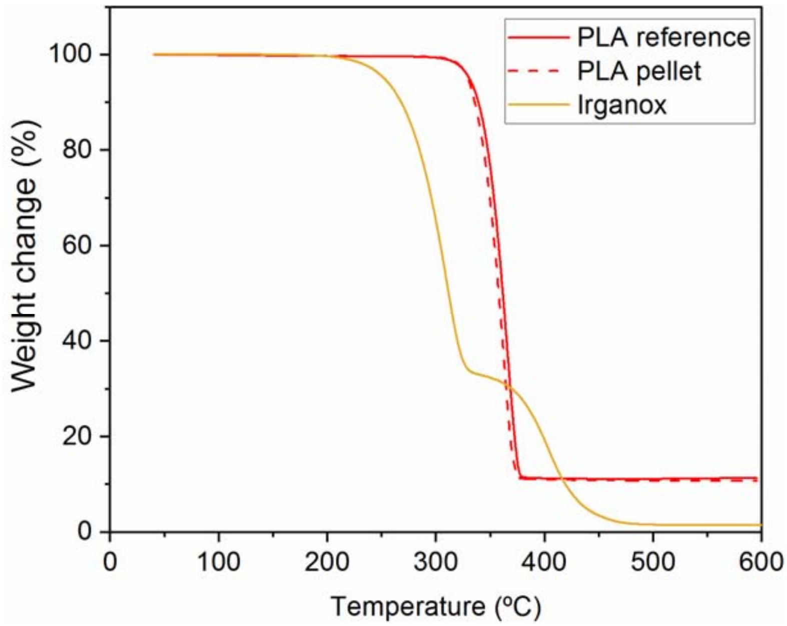

Figure S1. TGA of the reference PLA and PLA pellet and pure Irganox under inert atmosphere.

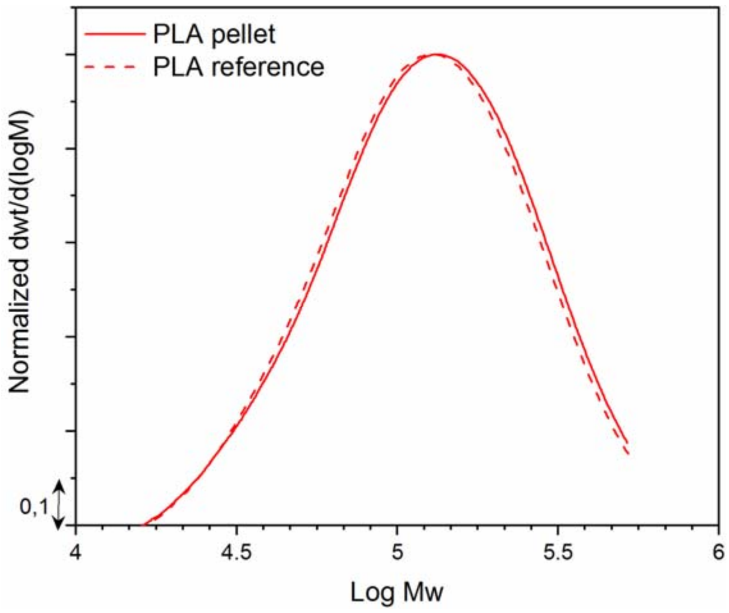

Figure S2. Molecular weight distributions of reference PLA and PLA pellet.

Table S1. MARHE values according to the EN 45545-2 standard for R1 (interior vertical surfaces) and R7 (external surfaces) requirements and different hazard levels (HL).

Requirement set (used for)	Test method reference	Parameter and unit	Maximum or Minimum	HL1	HL2	HL3
R1 (IN1A; IN1B; IN1D; IN1E; IN4; IN5; IN6A; IN7; IN8; IN9B; IN11; IN12A; IN12B; IN14; EX4A; F5)	T02 ISO 5658-2	*CFE* kWm^{-2}	Minimum	20 a	20 a	20 a
	T03.01 ISO 5660-1: 50 kWm^{-2}	*MARHE* kWm^{-2}	Maximum	-	90	60
	T10.01 EN ISO 5659-2: 50 kWm^{-2}	$D_S(4)$ dimensionless	Maximum	600	300	150
	T10.02 EN ISO 5659-2: 50 kWm^{-2}	*VOF4* min	Maximum	1 200	600	300
	T11.01 EN 17084 Method 1 50 kWm^{-2}	CIT_G dimensionless	Maximum	1,2	0,9	0,75

R7 (IN6B; IN12C; EX1A; EX1C; EX3; EX4B; EX5; EX6A; EX7; EX8; EL3C)	T02 ISO 5658-2	*CFE* kWm^{-2}	Minimum	20 a	20 a	20 a
	T03.01 ISO 5660-1: 50 kWm^{-2}	*MARHE* kWm^{-2}	Maximum	–	90	60
	T10.04 EN ISO 5659-2: 50 kWm^{-2}	D_S max. dimensionless	Maximum	–	600	300
	T11.01 EN 17084 Method 1 50 kWm^{-2}	CIT_G dimensionless	Maximum	–	1,8	1,5
